# Microscale spatial analysis provides evidence for adhesive monopolization of dietary nutrients by specific intestinal bacteria

**DOI:** 10.1371/journal.pone.0175497

**Published:** 2017-04-10

**Authors:** Yusuke Nagara, Toshihiko Takada, Yuriko Nagata, Shoichi Kado, Akira Kushiro

**Affiliations:** 1 Microbiological Research Department, Yakult Central Institute, Tokyo, Japan; 2 Safety Research Department, Yakult Central Institute, Tokyo, Japan; Instituto de Agroquimica y Tecnologia de Alimentos, SPAIN

## Abstract

Each species of intestinal bacteria requires a nutritional source to maintain its population in the intestine. Dietary factors are considered to be major nutrients; however, evidence directly explaining the *in situ* utilization of dietary factors is limited. Microscale bacterial distribution would provide clues to understand bacterial lifestyle and nutrient utilization. However, the detailed bacterial localization around dietary factors in the intestine remains uninvestigated. Therefore, we explored microscale habitats in the murine intestine by using histology and fluorescent *in situ* hybridization, focusing on dietary factors. This approach successfully revealed several types of bacterial colonization. In particular, bifidobacterial colonization and adhesion on granular starch was frequently and commonly observed in the jejunum and distal colon. To identify the bacterial composition of areas around starch granules and areas without starch, laser microdissection and next-generation sequencing-based 16S rRNA microbial profiling was performed. It was found that *Bifidobacteriaceae* were significantly enriched by 4.7 fold in peri-starch areas compared to ex-starch areas. This family solely consisted of *Bifidobacterium pseudolongum*. In contrast, there was no significant enrichment among the other major families. This murine intestinal *B*. *pseudolongum* had starch-degrading activity, confirmed by isolation from the mouse feces and *in vitro* analysis. Collectively, our results demonstrate the significance of starch granules as a major habitat and potential nutritional niche for murine intestinal *B*. *pseudolongum*. Moreover, our results suggest that colonizing bifidobacteria effectively utilize starch from the closest location and maintain the location. This may be a bacterial strategy to monopolize solid dietary nutrients. We believe that our analytical approach could possibly be applied to other nutritional factors, and can be a powerful tool to investigate *in vivo* relationships between bacteria and environmental factors in the intestine.

## Introduction

The gut microbiota are known to modulate host health and diseases including metabolic syndrome, inflammatory bowel disease, and cancer [1], and modulation of gut microbiota is now expected to be a promising approach in the prevention or treatment of diseases. Bacterial composition varies among individuals and is influenced by several factors, particularly diet [2]; however, current knowledge does not adequately explain the mechanisms involved in the variability of bacterial compositions. The bacterial activities underlying community shifts are still unclear, and firm control of microbiota by intervention is a challenge. More clarity regarding microbial ecology in the intestine will provide clues for developing methods to control the microbiota.

Indigenous bacteria are known to successfully maintain their numbers in the intestinal microbiota; hence, they are presumed to have established an ecological or nutritional niche that enables them to thrive. Information regarding what nutrients are directly utilized by which bacteria has accumulated through *in vitro* studies. However, studies have shown that nutrients identified *in vitro* sometimes fail to expand the target organisms or also expand non-targeted organisms when fed to humans or test animals [3]. Thus, information is still limited regarding the degradation processes of dietary macromolecules, which end up with utilization by specific bacteria among the various intestinal bacteria.

To elucidate the niche of organisms, it is important to understand their spatial distribution, which may reflect their required environmental factors. In particular, detailed information on bacterial lifestyle will be provided by analyses with an appropriate spatial resolution that corresponds to the activities of target organisms. In intestinal microbiology, several groups have analyzed and reported microscale spatial organizations of microbiota [4–7], and a few reports have documented bacterial localization around dietary residues [8–10]. These studies revealed associations of several bacterial groups with dietary or particulate structures, and highlighted that some intestinal bacteria have microscale habitats that may reflect their nutrient demands. However, the components of individual dietary structures have neither been precisely specified from the fecal material nor from the intestinal content, and the bacterial composition around each structure has not been fully described. The utilization of components in the structures by colonizers also remains unclear, even though it is important for understanding the role of each structure in colonization. The aim of this study was to explore the microscale localization of bacterial groups around specific dietary nutrients, and to determine the metabolic and ecological relationship between each nutrient and the surrounding colonizers. Results indicate that a specific bacteria colonized onto a single dietary factor, proposing a bacterial strategy to monopolize the dietary nutrient.

## Materials and methods

All animal experiments were performed according to the guidelines of the Yakult Central Institute and were approved by the Animal Experimental Committee of Yakult Central Institute.

### Animals

C57BL/6J Jcl mice (6 weeks old) were purchased from CLEA Japan (Tokyo, Japan) and maintained in a specific pathogen-free facility. All mice were fed CE-2 chow (CLEA Japan, Tokyo) by the breeder before purchase, and then fed MF chow (Oriental Yeast, Tokyo, Japan) and water ad libitum after purchase. The mice were anesthetized with sodium pentobarbital and sacrificed after 5 weeks, on Day 35 or 36 (the day of purchase was considered to be Day 0), at 11 weeks of age. Animals were monitored daily and weighed twice a week. No individual animal became ill or died.

Two batches of animals were used in this study. Batch #1 with 4 mice were subjected to microscopic observation and laser microdissection (LMD), and batch #2 with 6 mice was used for bacterial culture and fecal microbiota analysis. Batch #2 was also subjected to microscopic observation and LMD, except for two mice which were used for preparatory experiment for LMD. Consequently, the sections of 8 mice were subjected to LMD analyses, and sections from 6 mice were successfully analyzed (described later). According to the manufacturer, major starch sources of MF chow are corn and wheat (mainly bran), and standard composition (w/w) of MF chow is as follows; 7.9% moisture, 23.1% crude protein, 5.1% crude fat, 5.8% crude ash, 2.8% crude fiber, 55.3% Nitrogen-free extract.

### Preparation of paraffin sections

The whole gastrointestinal tract with digesta was fixed using methacarn solution (methanol/chloroform/acetic acid = 6:3:1) for 15–60 min or modified Carnoy’s solution (ethanol/chloroform/acetic acid = 6:1:6) [5] for 24 h. The intestinal parts filled with digesta were collected and cut into approximately 5–8-mm long sections. Trimmed tissues were first embedded in agarose, as previously described [6], and were then paraffin-embedded; thereafter, 4-μm sections of the paraffin-embedded blocks were cut for microscopic analysis, and 10-μm sections were cut for LMD.

### FISH and other staining

FISH was performed as previously described [11]. The FISH probes used in this study are summarized in S1 Table in the supplemental material. Sections were deparaffinized and incubated with each FISH probe in a hybridization buffer (4.5 ng/μL probe, 750 mM NaCl, 100 mM Tris-HCl, 5 mM EDTA, 0.01% BSA, 10% sodium dextran sulfate, pH 8.0) at 40°C for 6 h or overnight. The specimens were then washed for 20 min in a wash buffer (50 mM NaCl, 4 mM Tris-HCl, 0.02 mM EDTA, pH 7.5) at 45°C and mounted using VECTASHIELD with DAPI (Vector Laboratories, Burlingame, CA, USA).

For starch staining, after the FISH washing step, a drop of Lugol's solution (#88031, Muto Pure Chemicals, Tokyo, Japan) was placed onto sections. After 30–60 s, the slide was washed briefly with water and dried immediately. Purple-stained starch granules were confirmed before and after mounting. Once mounted, specimens were observed within minutes, before Lugol's staining disappeared. Solutions for Alcian blue-PAS staining were purchased from Muto Pure Chemicals. For co-staining, FISH was performed after Alcian blue-PAS staining.

Fluorescence microscopy images were captured by assigning pseudocolors to acquired monochrome images at each wavelength.

### Laser microdissection

A part of the distal colon with digesta was subjected to LMD using a PALM MicroBeam IV system (Carl Zeiss Microscopy GmbH, Jena, Germany). Colon sections of 8 mice were first prepared, and sections of single mouse was omitted, since they had no intestinal content. Ten-micrometer paraffin sections were stained with Lugol’s solution and briefly washed with water. Because sections were observed without FISH staining, bacterial accumulation and taxonomy were indistinguishable during sample collection. Purple-stained starch granules and the surrounding regions (40-μm diameter) were excised from sections and collected into an AdhesiveCap 500 polymerase chain reaction (PCR) tube (Carl Zeiss Microscopy GmbH, Jena, Germany) as peri-starch samples. All of the observed starch granules in each section were collected, except for those present as chunks within intact plant tissue. We judged these granules as inadequate for analysis because FISH analysis confirmed that there were no or few bacteria in intact plant tissues that contained chunks of starch. In parallel, areas without starch granules were randomly selected and collected as ex-starch samples. Similar amount was collected for both groups; 3.5–6.0 x 10^4^ μm^2^ (approximately 25–50 regions from 2–3 sections) for each peri-starch sample, 1.0–2.0 x 10^5^ μm^2^ for each ex-starch sample.

### 16S rRNA microbial profiling

DNA was extracted from LMD samples by using the DNeasy Blood & Tissue Kit (Qiagen, Venlo, Netherlands) according to the manufacturer’s instructions. As previously described [12–14], the V1-V2 region of the 16S rRNA gene was amplified from the extracted DNA by using 27Fmod2-MiSeq and 338R-MiSeq primers which contain Golay barcode and Illumina adapter sequences (S1 Table). SYBR Premix Ex Taq II (Tli RNaseH Plus) (Takara Bio, Shiga, Japan), and 7500 Real-Time PCR System (Thermo Fisher scientific, Waltham, MA, USA) were used for amplification. PCR cycle was as follows; 50°C for 2 min, 95°C for 10 min, and a repeated cycle of 95°C for 30 sec—55°C for 30 sec—72°C for 90 sec. According to the previous studies [13, 14], PCR was monitored by SYBR signal, and stopped before signal saturation to minimize bias and erroneous product. Amplification was not successful for a sample that was fixed by modified Carnoy’s solution for 24 h. This sample was excluded from the following analysis, and remaining samples from 6 mice were analyzed. The barcoded PCR products were purified using Agencourt AMPure XP (Beckman Coulter, Brea, CA) and measured by Quant-iT PicoGreen dsDNA Assay Kit (Thermo Fisher Scientific, Waltham, MA, USA). The Miseq library was constructed by mixing equal amount of DNA for every sample, and analyzed using MiSeq Reagent Kit v2 (Illumina, San Diego, CA, USA). Resultant sequence data were analyzed by QIIME 1.9 [15], as previously described [13, 14]. In brief, raw paired-end reads were joined (fastq-join, 120 bp of minimum overlap is required and maximum 8% mismatch is allowed) and quality filtered (minimum quality score 25). OTUs were picked by USEARCH [16] with a threshold of 97%, and chimera were removed using GOLD database as reference database [17]. The most abundant representative sequence was picked from each OTU and taxonomy was assigned using RDP method [18] and greengenes 13_8, a default database equipped in QIIME, and bacterial composition was calculated at family level. The representative sequences were also analyzed by local BLASTn (http://blast.ncbi.nlm.nih.gov/) using database of The All-Species Living Tree Project [19] to determine the closest species. Threshold of 97% was employed for identification of species. OTUs which consist less than 0.005% of total reads were filtered out according to Bokulich et al. [20], and at least 39,000 reads were analyzed for every sample.

### Design of species-specific FISH probes

The Ribosomal Database Project website (http://rdp.cme.msu.edu/) was used to obtain reference 16S rRNA gene sequences of type strains of *Bifidobacterium*. The sequences were aligned, and sequence motifs specific for *B*. *pseudolongum* subsp. *globosum* and *B*. *pseudolongum* subsp. *pseudolongum* were selected. The designed Bpl190 probe (S1 Table) was examined for specificity of hybridization by using the reference bacterial strains listed in S2 Table. The reference strains were cultured with optimal broth, fixed in 4% paraformaldehyde, and analyzed by FISH.

### Culture of murine indigenous bifidobacteria

Fresh fecal samples were collected on Days 1 and 16. These samples were suspended in PBS and plated onto 5% horse blood-supplemented BL agar and CPLX agar [21]; these plates were incubated anaerobically for 2–3 days at 37°C. Colonies were selected by Gram staining and cellular morphology, and inoculated in GAM broth (#05422, Nissui Pharmaceutical, Tokyo, Japan) supplemented with 1% glucose, or modified GAM agar (#05426, Nissui Pharmaceutical, Tokyo, Japan). Subsequently, cultured bacteria were examined by FISH by using a *Bifidobacterium*-specific probe, Bif153 (S1 Table). Single colonies were picked and streaked three times to obtain pure cultures, and the isolates were stored at -80°C.

### Evaluation of starch degradation

Isolates were anaerobically cultured on modified GAM agar (0.5% (w/v) soluble starch is contained in the product). Plates were flooded with 1:1-diluted Lugol's solution for approximately 30 s. Subsequently, Lugol's solution was removed and starch degradation was evaluated by formation of a clear zone around the colonies.

### Analysis of 16S rRNA gene sequence and taxonomical identification

Isolates were cultured on modified GAM agar and harvested. Genomic DNA was extracted as previously described [22]. The 16S rRNA gene was amplified with 27F and 1522R primers (S1 Table) using Takara ExTaq (Takara Bio, Shiga, Japan). The sequences of the amplified product were analyzed with 27F and 520R primers using BigDye3.1 and ABI3100 systems (Thermo Fisher Scientific, Waltham, MA, USA).

## Results

### Overview of murine intestinal digesta

**Fig 1:**
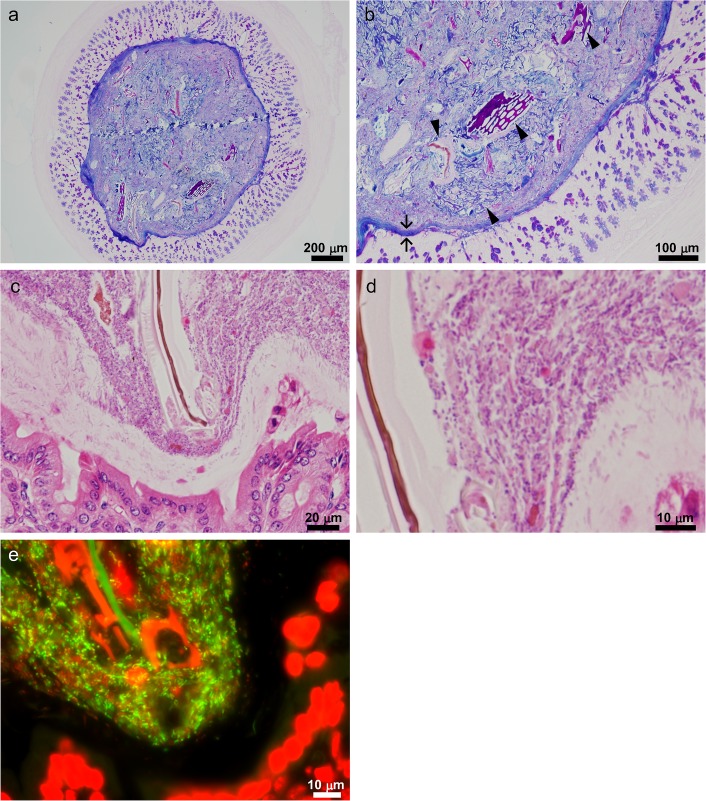
Murine intestinal tract filled with indigenous bacteria and food residue. (a–e) Cross section of murine colon and digesta. (b) and (d) are magnified images of (a) and (c), respectively. (b) Mucus layer surrounding the mucosa (arrow) and food residues, including plant tissues (arrowhead), in the digesta were visualized by Alcian blue-PAS staining. (d) Intestinal bacteria were visualized by hematoxylin-eosin staining. (e) Structures stained with hematoxylin-eosin were labeled by FISH using a universal bacterial probe Eub338 (green) and DAPI (red). Images are representative of at least 3 individual mice. Bars = 200 μm (a), 100 μm (b), 20 μm (c), 10 μm (d, e).

**Fig 2:**
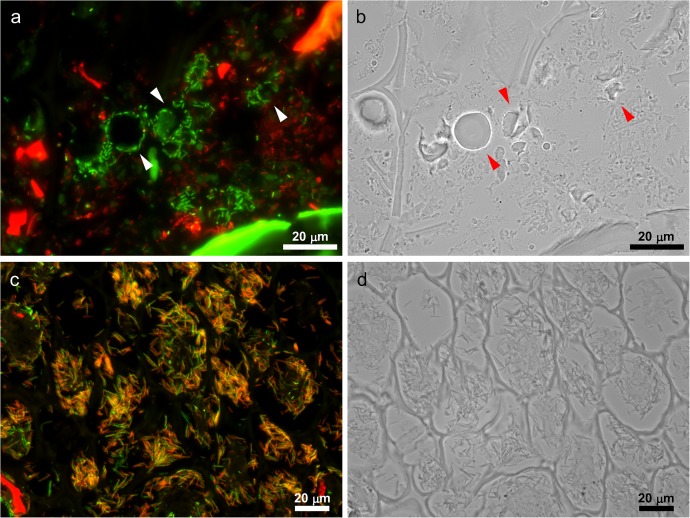
Structures colonized by bacteria in the digesta. (a,b) Cross section of murine jejunum stained by Eub338 (green) and DAPI (red). Bacteria accumulate around granular structures with a diameter of approximately 5–15 μm (arrowhead). (c,d) Cross section of murine cecum stained by Eub338 (green) and DAPI (red). Inner area of plant cell wall was filled with bacteria. Images are representative of at least 7 individual mice. (a,c) Fluorescence microscopy. (b,d) Bright-field images. Bars = 20 μm.

**Fig 3:**
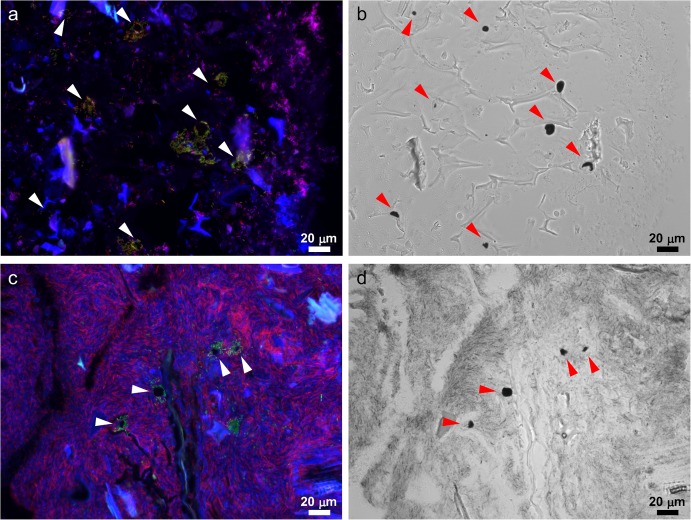
*Bifidobacterium* accumulated onto starch granules in broad regions of the murine intestinal tract. (a, b) Cross section of murine jejunum stained by Bif153 (green), Eub338 (red), Lab158 (blue), and Lugol’s solution. Bif153-positive bifidobacteria preferentially colonized onto starch granules (arrowhead) in the presence of jejunal-dominant lactic acid bacteria. (c,d) Cross section of murine colon stained by Bif153 (green), Erec482 (red), Clept1240 (blue), and Lugol’s solution. Similar to the jejunum, Bif153-positive bifidobacteria preferentially colonized starch granule surfaces (arrowhead) in the presence of colonic-dominant bacteria, *Clostridium* cluster XIVa and XIVb and *Clostridium leptum* subgroup. Images are representative of at least 7 individual mice. (a,c) Fluorescence microscopy. (b,d) Bright-field microscopy. Bars = 20 μm.

To explore the bacterial microscale localization, the intestinal contents of conventionally raised mice were paraffin-embedded, and sections were analyzed using several staining methods. In these preparations, the overall structure of the digesta was maintained. Alcian blue-periodic acid-Schiff (PAS) staining revealed components of the intestinal digesta and their distribution (Fig 1A and 1B). Plant cell wall-like structures were frequently observed with heterogeneous Alcian blue-PAS staining, and a mucus layer was observed to surround the intestinal digesta (Fig 1A and 1B). Bacteria were visualized clearly by hematoxylin-eosin staining, a bacterial universal FISH probe (Eub338), and DAPI (Fig 1C–1E). The lumen of the small intestine had a large unoccupied area (Figs 2A, 2B, 3A and 3B), whereas most of the colonic luminal area was filled with bacteria and dietary residues (Fig 1, also see Fig 3C and 3D).

### Indigenous bacteria colonize onto starch granules

FISH analysis of the intestinal digesta revealed several types of bacterial localization. For example, rod-shaped bacteria accumulated onto granular structures (Fig 2A and 2B), and many fusiform bacteria colonized inside the plant cell wall (Fig 2C and 2D). Among these types of localization, the accumulation onto granules was the most prominent type. Many bacteria adhered to the granules. These granules had a typical diameter of 5–15 μm and presented a cracked appearance (Fig 2B).

To identify the bacteria-enriched granules, several staining methods were applied. Alcian blue-PAS and FISH co-staining indicated that the granules were PAS-positive but Alcian blue-negative (S1 Fig). This suggests that the granules contained carbohydrates and were neutral or basic.

The chow used in this study contained maize and wheat, which both mainly consist of starch. Starch is a neutral carbohydrate, with a diameter of 5 to 20 μm [23]. Some starch granules are known to have a crack, similarly to the bacteria-colonized granules. Therefore, we examined whether the observed granules were starch or not by staining the sections with Lugol’s iodine. The granules were stained purple (Fig 3B and 3D), indicating that they were starch granules.

### Bifidobacteria colonize starch granules

To identity the bacteria surrounding the starch granules, the intestinal sections were examined by FISH using several group-specific probes. The starch-colonizing bacteria were specifically stained with a bifidobacteria probe (Bif153), but not with probes for other dominant bacteria; lactobacilli in the jejunum and *Clostridium* cluster XIVa, XIVb and *Clostridium leptum* subgroup in the colon (Fig 3). Colonization was detected in the jejunum, distal colon, (Fig 3) and feces (S2 Fig).

### Accumulation around starch granules is specific to *Bifidobacterium pseudolongum*

**Fig 4:**
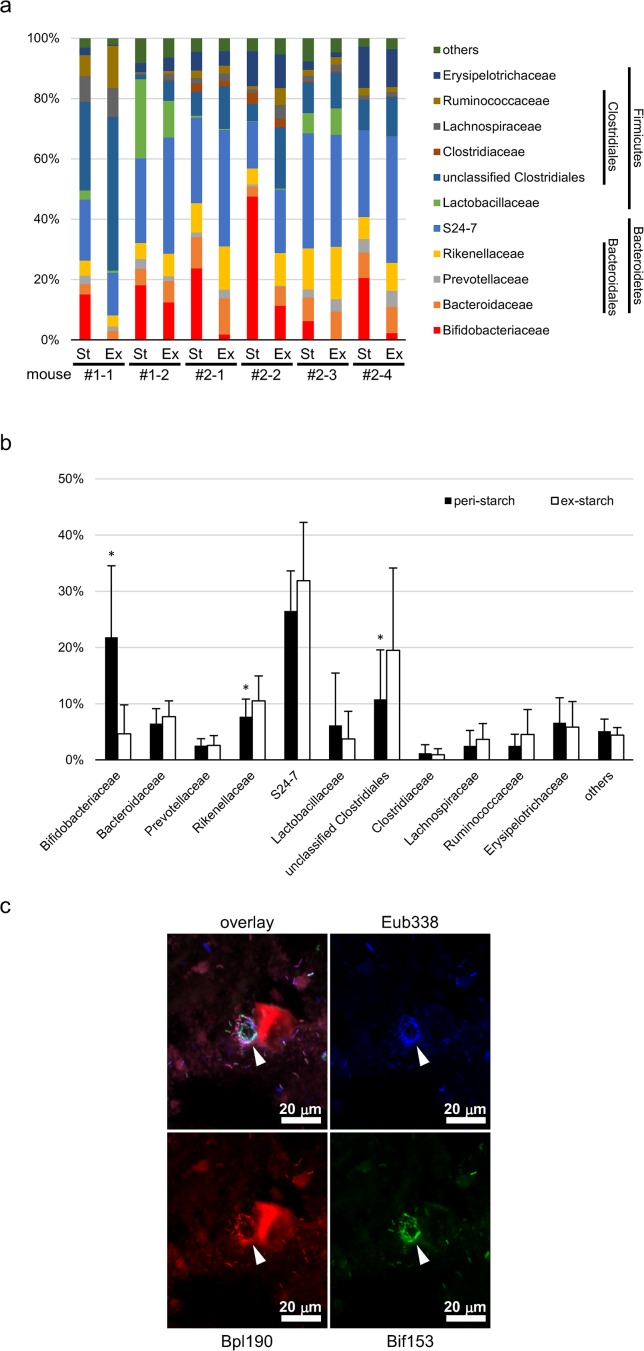
*Bifidobacterium pseudolongum* accumulates onto dietary starch granules in a species-specific manner. (a,b) Intestinal contents in the peri-starch areas (St) and ex-starch areas (Ex) were collected from sections of the murine colon using LMD and subjected to NGS-based 16S rRNA microbial profiling. The results of 6 mice were shown at the family level. (b) Mean ± SD is shown. *; significantly different between peri-starch and ex-starch (p<0.05, two-tailed paired t-test). (c) *B*. *pseudolongum* around a starch granule stained by Bpl190 (red), Bif153 (green), and Eub338 (blue) (arrowhead). Images are representative of 3 individual mice. Bars = 20 μm.

To quantitatively and comprehensively characterize the local bacterial composition around the starch granules, LMD and next-generation sequencing (NGS)-based 16S rRNA microbial profiling was performed. First, starch granules were visualized by Lugol’s staining, and then areas surrounding starch (peri-starch areas) and areas without starch (ex-starch areas) were randomly sampled (S3 Fig). Major bacterial families (comprising more than 1% of total number of reads) were commonly detected from LMD and fecal samples, though populations of several families including *Erysipelotricaceae* and *Lactobacillaceae* showed some difference between two sample sets. The difference is probably due to the different DNA extraction methods employed for each sample set. The results support overall adequacy of LMD-NGS analysis and therefore we considered comparison of bacterial population between two LMD samples will not be disturbed (Fig 4A and S4 Fig). The population of *Bifidobacteriaceae* showed, on average, a 4.7-fold significant enrichment in peri-starch areas compared to ex-starch areas (21.8% vs. 4.6%, n = 6, p<0.05, two-tailed paired t-test), consistent with the preceding FISH analysis. In contrast, populations of other major families (comprising more than 1% of total number of reads) did not show significant enrichment around starch (Fig 4B), and we observed a 0.6-fold reduction for unclassified *Clostridiales* and a 0.7-fold reduction for *Rikenellaceae* (10.8% vs. 19.5% and 7.7% vs. 10.5%, respectively, n = 6, p<0.05, two-tailed paired t-test).

Subsequently, OTU representative sequences were analyzed by BLAST to determine the taxonomy of bacteria which belong to the enriched or excluded family. It revealed that all OTUs assigned to *Bifodobacteriaceae* in both peri-starch and ex-starch areas were *B*. *pseudolongum* (i.e., *B*. *pseudolongum* subsp. *pseudolongum* or *B*. *pseudolongum* subsp. *globosum*; these two subspecies could not be distinguished by this analysis and are henceforth collectively referred to as *B*. *pseudolongum*). Regarding the NGS data, we double-checked at the species-level that the starch-surrounding bacteria were indeed *B*. *pseudolongum* by using a FISH probe (Fig 4C), which was newly developed for this study (Bpl190; S1 and S2 Tables). None of other OTUs assigned to unclassified *Clostridiales* nor *Rikenellaceae* were identified to species level.

### Isolated murine *B*. *pseudolongum* showed amylolytic activity

**Fig 5:**
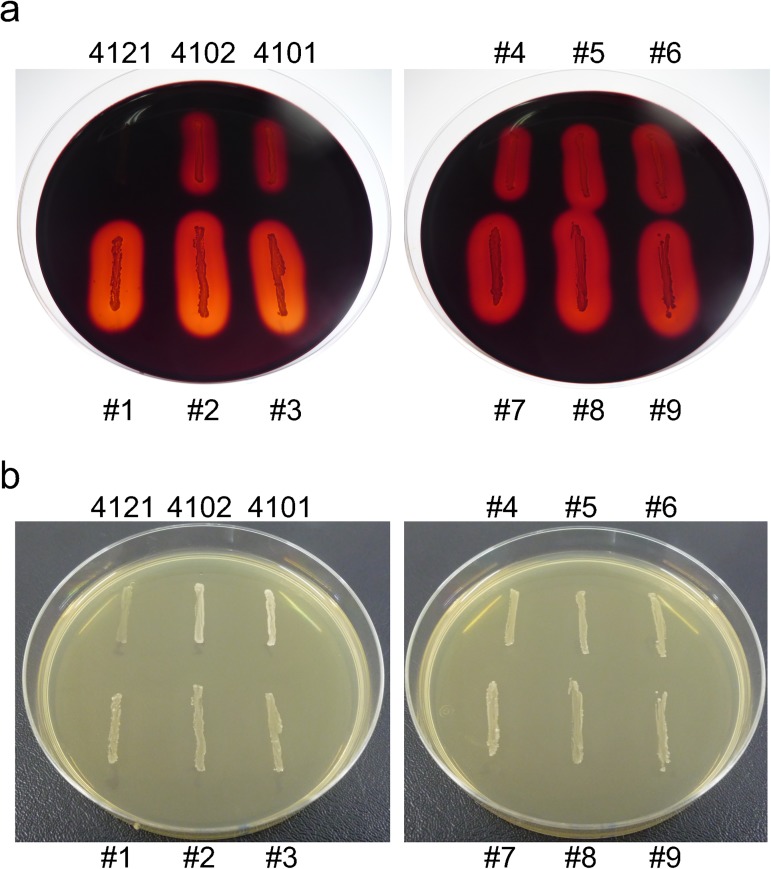
The isolated murine *Bifidobacterium pseudolongum* shows amylolytic activity. (a) A modified GAM agar plate was stained by Lugol’s solution. Starch in agar was degraded around colonies. (b) Before staining. 4121; *B*. *animalis* subsp. *lactis*, type strain YIT 4121, 4102; *B*. *pseudolongum* subsp. *pseudolongum*, type strain YIT 4102, 4101; *B*. *pseudolongum* subsp. *globosum*, type strain YIT 4101, #1–9; Isolates identified as *B*. *pseudolongum*. (See Table 1).

To examine whether the colonized *B*. *pseudolongum* can utilize starch, we isolated bifidobacteria from murine feces and evaluated their amylolytic activity. Twenty-two colonies were isolated and confirmed as bifidobacteria using the Bif153 probe. All colonies were defined as a single strain by random-amplified polymorphic DNA (RAPD) analysis (performed as described by Akopyanz *et al*. [24] using primers listed in S1 Table). Among them, 9 isolates were used for verification. The 16S rRNA gene of all 9 isolates showed >99% identity with *B*. *pseudolongum* subsp. *pseudolongum* and *B*. *pseudolongum* subsp. *globosum*, confirming that we were able to isolate the colonizing species from the same mice (Table 1). Furthermore, these isolates were found to have amylolytic activity using the starch agar method (Table 1, Fig 5).

**Table 1:** Amylolytic activity of isolated *Bifidobacterium pseudolongum* strains and type strains.

Strain	Identified species	Amylolytic activity
**Type strain**		
YIT 4121^T^	*B*. *animalis* subsp. *lactis*	−
YIT 4102^T^	*B*. *pseudolongum* subsp. *pseudolongum*	+
YIT 4101^T^	*B*. *pseudolongum* subsp. *globosum*	+
**Isolate**		
#1	*B*. *pseudolongum*	+ +
#2	*B*. *pseudolongum*	+ +
#3	*B*. *pseudolongum*	+ +
#4	*B*. *pseudolongum*	+
#5	*B*. *pseudolongum*	+ +
#6	*B*. *pseudolongum*	+ +
#7	*B*. *pseudolongum*	+ +
#8	*B*. *pseudolongum*	+ +
#9	*B*. *pseudolongum*	+ +

++; strong, +; positive, −; negative.

## Discussion

Previous studies have analyzed the microscale localization of intestinal bacteria and revealed distribution patterns unique for bacterial groups. However, these studies mainly focused on the community around the mucosal surface, and to our knowledge, the bacterial composition around individual dietary factors has not been studied *in vivo*. The significance of microscale colonization in bacterial nutrient acquisition also remains uninvestigated, probably because of the lack of tools to identify each dietary factor under a microscope, and the difficulty in isolating the strains involved in colonization of nutrients. In the present study, we were able to successfully obtain a novel picture of microbial behavior in the intestine by using plural lines of methods. Starch was identified by staining of serial sections with multiple methods. By combining LMD and NGS-based community profiling, we could determine the bacterial composition in small areas. This analysis provided species-level information that helped us to isolate the colonizing species.

At least two types of microscale bacterial colonization onto dietary factors were discovered in this study: bifidobacterial accumulation onto starch granules and accumulation of fusiform bacteria in plant tissue. This reinforces the notion that bacteria are not homogenously distributed in the intestinal content but have their own microscale habitats [5, 9]. This observation implies that a fraction of bacteria effectively utilizes nutrients by localizing around the nutrients.

One of the most prominent colonizations observed was the colonization of starch granules by *B*. *pseudolongum*, which was consistently detected by co-staining with FISH and Lugol’s iodine, and LMD analyses. It is known that some bifidobacteria, including *B*. *pseudolongum*, adhere to granular starch in *in vitro* pure-culture or anaerobic continuous-flow culture of fecal inocula [10, 25, 26]. However, the occurrence and quantity of the adhesion in the intestine has not been determined. An electron microscopic analysis suggested similar adhesion *in vivo*; however, the taxonomy of adherent bacteria was not determined with strong evidence in the report [27, 28]. In this study, we were able to demonstrate that bifidobacteria adhere to starch granules not only *in vitro*, but also in the intestinal environment where hundreds of other types of bacteria are living together.

In FISH analysis, only bifidobacteria were detected as starch-colonizing bacteria, and other major bacterial groups showed no association with starch. This bifidobacterial species selectivity was also demonstrated by comparing the microbial composition of peri-starch areas and other areas using LMD and NGS. Furthermore, the adhesion was intense and observed consistently in the jejunum, distal colon, and feces. This suggests that the adhesion is maintained from the upper to the lower intestinal tract with strong affinity. These multiple pieces of evidence raise the possibility that this adhesion is a bacterial strategy for exclusive monopolization of favorable nutrients; the colonizing bifidobacteria effectively utilize the nutrients from the closest location, and restrict utilization by other bacteria by occupying the surface of the nutrient. This hypothesis is substantiated by amylolytic activity of a *B*. *pseudolongum* strain isolated from the same mice. However, in the present study, starch-degrading ability was not confirmed experimentally, regarding bacteria other than bifidobacteria (i.e. potential competitors of bifidobacteria). Therefore, this hypothesis should be further examined in future studies. It will be useful to phenotypically identify and analyze localization of potential competitors which are sharing the same environment with bifidobacteria.

This idea might partly explain the observation of a bifidobacterial response on starch intake in previous reports (i.e., specific expansion of bifidobacteria and increase in short-chain fatty acids [29–31]). Previous genome analyses also have explained these points from a different point of view. It was described that several bifidobacterial strains have starch degradation pathway genes, which are induced by starch. This suggests that starch is a preferable carbon source for *Bifidobacterium* [32, 33]. Multiple reports have described that bifidobacteria utilize starch *in vitro* [26, 34–36]. However, such activity has not been shown in the intestine, and it is unknown whether large starch granules have a direct and specific effect on bifidobacteria, or whether pre-degradation into dextran or malto-oligosaccharide by other bacteria is necessary. Our results clarified that starch directly affects bifidobacteria *in vivo*, at least by adhesion, and imply a degradation process independent of other bacteria.

This study identified only one bifidobacterial species, *B*. *pseudolongum*, which is a common species in the intestine of animals including rodents [37]. However, it is likely that other species or other families of bacteria colonize onto starch granules in other systems such as the human or mouse intestine with different bacterial compositions. Consistent with this possibility, the bifidogenic effects of starch were variable in previous human studies [38, 39]. The effects may depend on whether or not each individual has dominant bacteria with the ability to capture and effectively utilize starch granules, and whether or not the bacteria belong to bifidobacteria. It has been reported that several species of *Bifidobacterium*, *Bacteroides*, and other species including *Eubacterium rectale* and *Ruminococcus bromii* are major human colonic bacteria with starch-utilizing ability [34–36, 40], although OTUs assigned to *E*. *rectale* or *R*. *bromii* were not detected in this study. Among these starch-utilizing bacteria, adhesion to starch granules has been evaluated among major bifidobacterial species [25]. Adhesive machinery has also been revealed in *Bacteroides thetaiotaomicron* [41], *E*. *rectale* [42], and *R*. *bromii* [43], although the mechanistic correspondence between molecular binding to starch and cellular attachment onto starch granules should be confirmed. Other than bifidobacteria, our experiment showed that the fraction of *Bacteroidaceae*, a family that contains many amylolytic species, was slightly larger in ex-starch areas compared to peri-starch areas. Similarly, the fractions of *Rikenellaceae* and an unidentified clade belonging to *Clostridiales* were significantly larger in ex-starch areas. Unfortunately, we were not able to identify the species in these families and for most of other OTUs, because of the low sequence identity. This is due to the lack of registered sequences for murine bacterial species in current reference database. More information on biochemical characteristics of murine intestinal bacterial species is necessary for detailed and precise discussion on how certain bacteria were excluded from peri-starch area. In general, bacteriocin-like substances or antibacterial metabolites can serve as mediators of such exclusion. Wang et al. reported that several species or clades of human colonic bacteria, in particular *Bacteroides* and *Fusobacterium*, can degrade only soluble or gelatinized starch but not granular starch, while *Bifidobacterium* can also degrade granular starch [36]. Similar limitation in utilizing ability might also be involved in the exclusion of certain bacteria. Processing or cooking, a common process which solubilize granular starch, might affect the responses of intestinal bacteria, because if starch is completely diffused, promoted utilization or monopolization by adhesion is impossible. Differences in the utilization of particulate and boiled starch have been discussed in detail by Ze et al. [43].

To our knowledge, this is the first *in vivo* report that describes species-specific localization of intestinal bacteria onto a single dietary factor and the relationship between bacterial localization and utilizing ability. Our analytical approach could possibly be applied to nutritional factors other than starch, and could be a powerful tool to investigate *in vivo* relationships between bacteria and environmental factors in the intestine. Further studies on the local microbial community around single dietary factors will be important, as metabolites or degradation products may be concentrated near the sites of colonization and affect the local community. Additionally, it remains unknown how adhesion onto nutrients affects the colonizer’s phenotype and influences the host physiology. These issues should be addressed in the future.

## Supporting information

S1 Fig PAS-positive, Alcian blue-negative granule with bacteria. A cross section of murine jejunum was stained by (a) Periodic acid-Schiff (PAS) and Alcian blue, and subsequently (b) stained by FISH (Eub338; green).
(PDF)

S2 Fig Bifidobacterial colonization onto starch in feces. A paraffin-embedded cross section of feces collected on Day 8 was stained by Bif153 (green), Lab158 (red), DAPI (blue), and Lugol’s solution. Bif153-positive bifidobacteria colonizing starch granules in feces, similar to that seen in the intestine. (a) Bright-field microscopy. (b) Fluorescent microscopy.
(PDF)

S3 Fig Areas collected by LMD. Colon content in peri-starch area (a, b) and ex-starch area (c, d) in sections were collected by LMD. Arrowhead in (a) indicates a starch granule stained purple by Lugol’s solution. The area surrounding starch granules (a) and the area in the rectangle in (c) were separately collected. Approximately 25–50 areas surrounding starch were collected to obtain at least 35,000 μm^2^, and subjected to DNA extraction and following analyses. (a,c) Before LMD. (b,d) After LMD.
(PDF)

S4 Fig Fecal bacterial composition determined by 16S rRNA microbial profiling. Fecal bacterial composition of six mice (#2–1 to #2–6) were examined from the beginning of feeding (day 0) until day 35, and shown at family level. Four of them (#2–1 to #2–4) were analyzed by LMD and results were shown in Fig 4.
(PDF)

S1 Table FISH probes and primers used in this study. (PDF)

S2 Table Validation of Bpl190 probe and reference strains used in this study. Validation of Bpl190 probe and reference strains used in this study. ++; strong, +; positive, −; negative, w; weak.
(PDF)
