# Supplementary material for: Microscale spatial analysis provides evidence for adhesive monopolization of dietary nutrients by specific intestinal bacteria
Source: PLoS One. 2017 Apr 10;12(4):e0175497. doi: 10.1371/journal.pone.0175497 (PMC5386278; doi:10.1371/journal.pone.0175497)
Supplement: S1 Fig — A cross section of murine jejunum was stained by (a) Periodic acid-Schiff (PAS) and Alcian blue, and subsequently (b) stained by FISH (Eub338; green). (PDF) [file pone.0175497.s001.pdf]

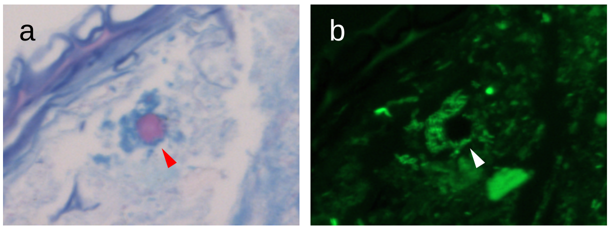

S1 Fig

PAS-positive, Alcian blue-negative granule with bacteria

A cross section of murine jejunum was stained by (a) Periodic acid-Schiff (PAS) and Alcian blue, and subsequently (b) stained by FISH (Eub338; green).
