# Supplementary material for: Microscale spatial analysis provides evidence for adhesive monopolization of dietary nutrients by specific intestinal bacteria
Source: PLoS One. 2017 Apr 10;12(4):e0175497. doi: 10.1371/journal.pone.0175497 (PMC5386278; doi:10.1371/journal.pone.0175497)
Supplement: S2 Fig — A paraffin-embedded cross section of feces collected on Day 8 was stained by Bif153 (green), Lab158 (red), DAPI (blue), and Lugol’s solution. Bif153-positive bifidobacteria colonizing starch granules in feces, similar to that seen in the intestine. (a) Bright-field microscopy. (b) Fluorescent microscopy. (PDF) [file pone.0175497.s002.pdf]

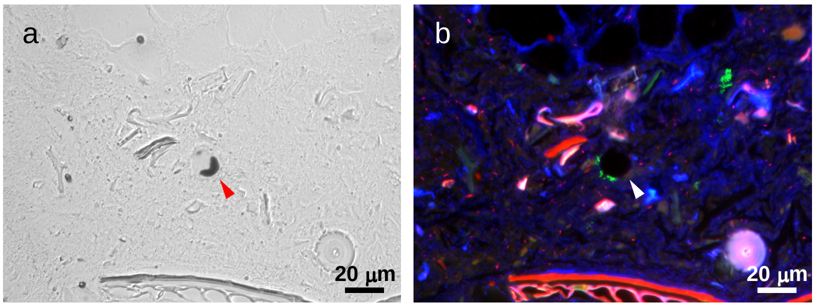

S2 Fig
Bifidobacterial colonization onto starch in feces
A paraffin-embedded cross section of feces collected on Day 8 was stained by Bif153 (green), Lab158 (red), DAPI (blue), and Lugol’s solution. Bif153-positive bifidobacteria colonizing starch granules in feces, similar to that seen in the intestine.
(a) Bright-field microscopy. (b) Fluorescent microscopy.
