# Supplementary material for: Microscale spatial analysis provides evidence for adhesive monopolization of dietary nutrients by specific intestinal bacteria
Source: PLoS One. 2017 Apr 10;12(4):e0175497. doi: 10.1371/journal.pone.0175497 (PMC5386278; doi:10.1371/journal.pone.0175497)
Supplement: S3 Fig — Colon content in peri-starch area (a, b) and ex-starch area (c, d) in sections were collected by LMD. Arrowhead in (a) indicates a starch granule stained purple by Lugol’s solution. The area surrounding starch granules (a) and the area in the rectangle in (c) were separately collected. Approximately 25–50 areas surrounding starch were collected to obtain at least 35,000 μm2, and subjected to DNA extraction and following analyses. (a,c) Before LMD. (b,d) After LMD. (PDF) [file pone.0175497.s003.pdf]

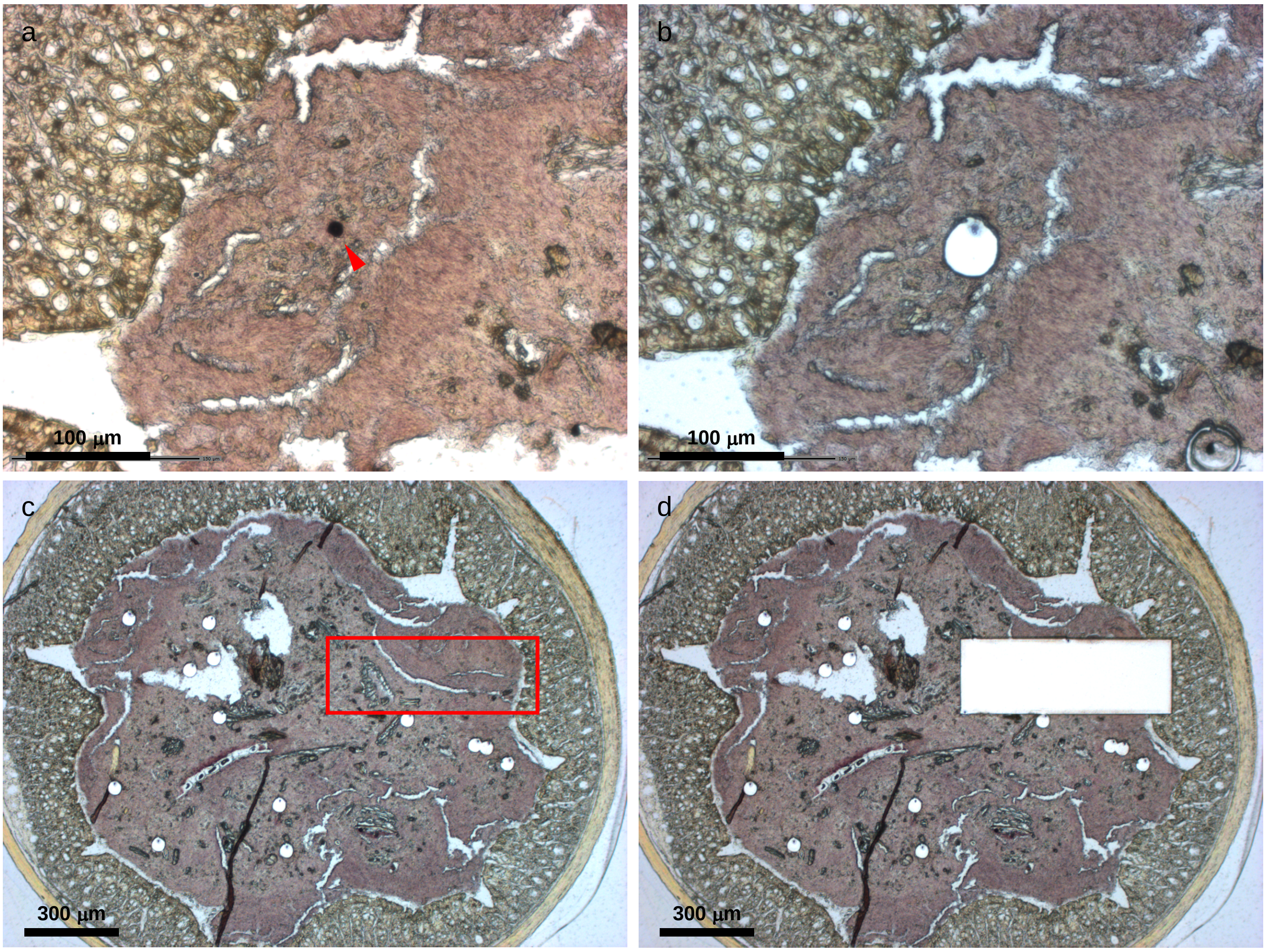

S3 Fig

Areas collected by LMD

Colon content in peri-starch area (a, b) and ex-starch area (c, d) in sections were collected by LMD. Arrowhead in (a) indicates a starch granule stained purple by Lugol's solution. The area surrounding starch granules (a) and the area in the rectangle in (c) were separately collected. Approximately 25-50 areas surrounding starch were collected to obtain at least 35,000 µm2, and subjected to DNA extraction and following analyses. (a,c) Before LMD. (b,d) After LMD.
