# Supplementary material for: Microscale spatial analysis provides evidence for adhesive monopolization of dietary nutrients by specific intestinal bacteria
Source: PLoS One. 2017 Apr 10;12(4):e0175497. doi: 10.1371/journal.pone.0175497 (PMC5386278; doi:10.1371/journal.pone.0175497)
Supplement: S4 Fig — Fecal bacterial composition of six mice (#2–1 to #2–6) were examined from the beginning of feeding (day 0) until day 35, and shown at family level. Four of them (#2–1 to #2–4) were analyzed by LMD and results were shown in Fig 4. (PDF) [file pone.0175497.s004.pdf]

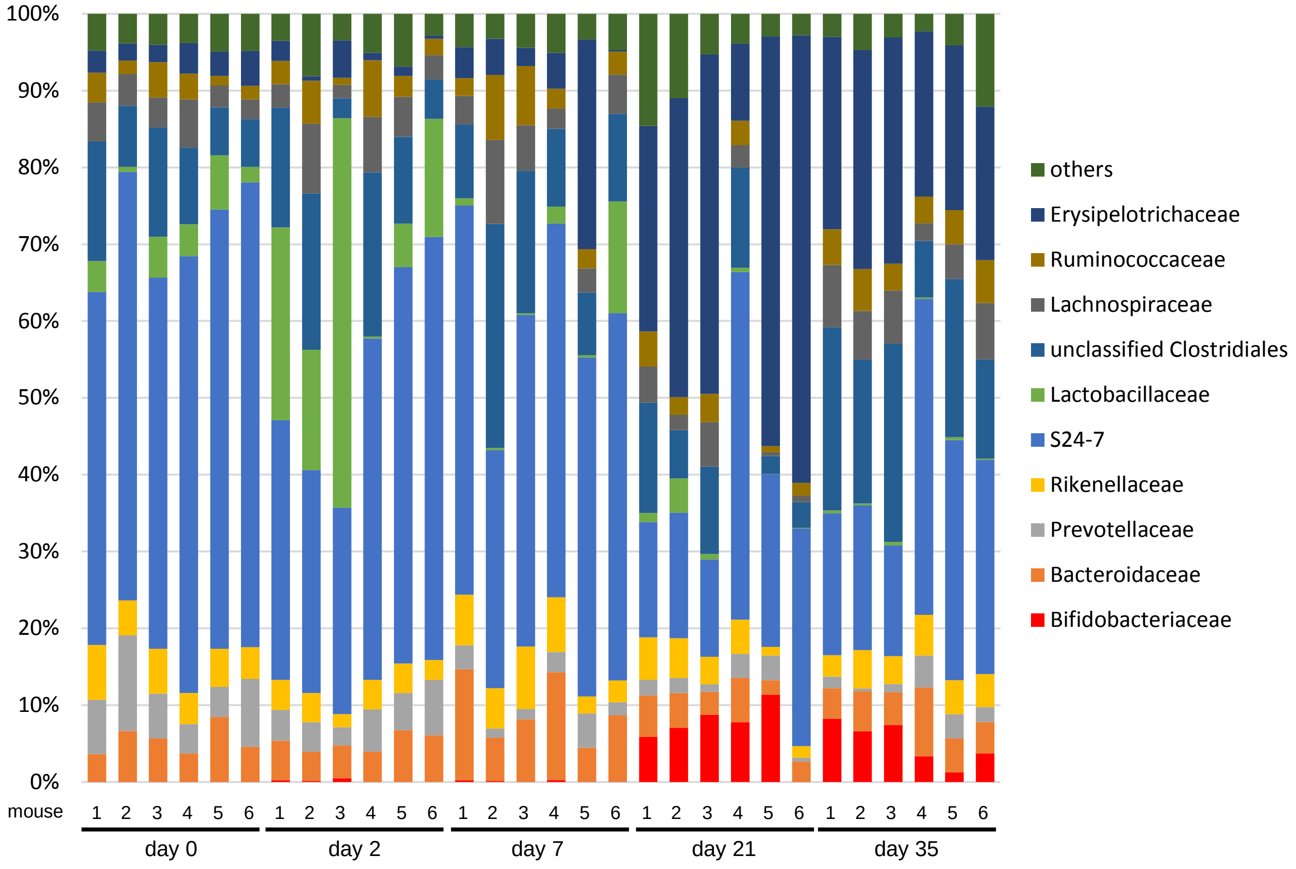

Figure S4
Fecal bacterial composition determined by 16S rRNA microbial profiling
Fecal bacterial composition of six mice (#2-1 to #2-6) were examined from the beginning of feeding (day 0) until day 35, and shown at family level. Four of them (#2-1 to #2-4) were analyzed by LMD and results were shown in Fig.4.
