# Supplementary material for: Microscale spatial analysis provides evidence for adhesive monopolization of dietary nutrients by specific intestinal bacteria
Source: PLoS One. 2017 Apr 10;12(4):e0175497. doi: 10.1371/journal.pone.0175497 (PMC5386278; doi:10.1371/journal.pone.0175497)
Supplement: S1 Table — (PDF) [file pone.0175497.s005.pdf]

Name	Sequence	Target	Fluorescent label	References
FISH probes				
Bpl190	CCACACTCGCATGCGCTCAT	*B. pseudolongum* group	FAM	This study
Eub338	GCTGCCTCCCGTAGGAGT	total bacteria	TAMRA	[1]
Bif153	ACCACCCGTTTCCAGGAG	*Bifidobacterium*	FITC+FAM or Cy5	[2]
Lab158	GGTATTAGCAYCTGTTTCCA	*Lactobacilli, Enterococci*	Cy5	[3]
Erec482	GCTTCTTAGTCARGTACCG	*Clostridium* cluster XIVa and XIVb	TAMRA	[4]
Clept1240	GTTTTRTCAACGGCAGTC	*Clostridium leptum* subgroup	Cy5	[5]
Primers				
16S metagenome	(underlined sequences hybridize to 16S rRNA gene)			
27Fmod2-MiSeq	AATGATACGGCGACCACCGAGATCTACACTCTTTCCCTACACGACGCTCTTCCGATCTAGRGTTYGATYMTGGCTCAG			-
338R-MiSeq	CAAGCAGAAGACGGCATACGAGAT-NNNNNNNNNNNN-GTGACTGGAGTTCAGACGTGTGCTCTTCCGATCTGCTGCCWCCCGTAGGWGT			-
RAPD				
Primer A	CCGCAGCCAA			[6]
Primer B	AACGCGCAAC			[7]
Primer E	GGCGTCGGTT			[7]
16S sequencing				
27F	AGAGTTTGATCMTGGCTCAG			[8]
1522R	AAGGAGGTGATCCARCCGCA			[9]
520R	ACCGCGGCTGCTGGC			[10]

S1 Table

FISH probes and primers used in this study.

References

1. **Amann RI, Binder BJ, Olson RJ, Chisholm SW, Devereux R, Stahl DA**. 1990. Combination of 16S rRNA-targeted oligonucleotide probes with flow cytometry for analyzing mixed microbial populations. Appl Environ Microbiol **56**:1919–1925.
2. **Takada T, Matsumoto K, Nomoto K**. 2004. Development of multi-color FISH method for analysis of seven Bifidobacterium species in human feces. J Microbiol Methods **58**:413–421.
3. **Harmsen H, Elfferich P**. 1999. A 16S rRNA-targeted Probe for Detection of Lactobacilli and Enterococci in Faecal Samples by Fluorescent In Situ Hybridization. Microb Ecol Health Dis **11**:3–12.
4. **Franks AH, Harmsen HJ, Raangs GC, Jansen GJ, Schut F, Welling GW**. 1998. Variations of bacterial populations in human feces measured by fluorescent in situ hybridization with group-specific 16S rRNA-targeted oligonucleotide probes. Appl Environ Microbiol **64**:3336–3345.
5. **Sghir A, Gramet G, Suau A, Rochet V, Pochart P, Dore J**. 2000. Quantification of bacterial groups within human fecal flora by oligonucleotide probe hybridization. Appl Environ Microbiol **66**:2263–2266.
6. **Akopyanz N, Bukanov NO, Westblom TU, Kresovich S, Berg DE**. 1992. DNA diversity among clinical isolates of Helicobacter pylori detected by PCR-based RAPD fingerprinting. Nucleic Acids Res **20**:5137–5142.
7. **Yuki N, Shimazaki T, Kushiro A, Watanabe K, Uchida K, Yuyama T, Morotomi M**. 2000. Colonization of the Stratified Squamous Epithelium of the Nonsecreting Area of Horse Stomach by Lactobacilli. Appl Environ Microbiol **66**:5030–5034.
8. **Lane DJ**. 1991. 16S/23S rRNA sequencing, p. 115–175. *In* Stackebrandt E and Goodfellow M (ed), Nucleic Acid Techniques in Bacterial Systematics. John Wiley & Sons: Chichester.
9. **Johnson JL**. 1994. Similarity analysis of rRNAs, p. 683–700. *In* Gerhardt P, Murray RGE, Wood WA, Krieg NR (ed), Methods for General and Molecular Bacteriology. American Society for Microbiology: Washington, D.C.
10. **Miyake T, Watanabe K, Watanabe T, Oyaizu H**. 1998. Phylogenetic analysis of the genus Bifidobacterium and related genera based on 16S rDNA sequences. Microbiol Immunol **42**:661–667.
