# Supplementary material for: Microscale spatial analysis provides evidence for adhesive monopolization of dietary nutrients by specific intestinal bacteria
Source: PLoS One. 2017 Apr 10;12(4):e0175497. doi: 10.1371/journal.pone.0175497 (PMC5386278; doi:10.1371/journal.pone.0175497)
Supplement: S2 Table — Validation of Bpl190 probe and reference strains used in this study. ++; strong, +; positive, −; negative, w; weak. (PDF) [file pone.0175497.s006.pdf]

Species	Strain	Detection	Species	Strain	Detection
B. pseudolongum subsp. *globosum*	YIT 4101^{T}	+	*B. bifidum*	YIT 4039^{T}	−
B. pseudolongum subsp. *globosum*	YIT 4041	+	*B. breve*	YIT 4014^{T}	−
B. pseudolongum subsp. *globosum*	YIT 10340	+	*B. catenulatum*	YIT 4016^{T}	−
B. pseudolongum subsp. *globosum*	YIT 10392	+	*B. choerinum*	YIT 4067^{T}	−/w
B. pseudolongum subsp. *globosum*	YIT 10393	+	*B. cuniculi*	YIT 4093^{T}	−/w
B. pseudolongum subsp. *pseudolongum*	YIT 4102^{T}	+	*B. galicum*	YIT 4085^{T}	−
B. pseudolongum subsp. *pseudolongum*	YIT 4040	+	*B. indicum*	YIT 4083^{T}	−
B. pseudolongum subsp. *pseudolongum*	YIT 4051	+	*B. longum*	YIT 4021^{T}	−
B. pseudolongum subsp. *pseudolongum*	YIT 4084	+	*B. pseudocatenulatum*	YIT 4072^{T}	−
B. pseudolongum subsp. *pseudolongum*	YIT 4103	+	*Bacteroides vulgatus*	YIT 6159^{T}	−
B. adolescentis	YIT 4011^{T}	−	*Eubacterium rectale*	YIT 6082^{T}	−
B. animalis subsp. *animalis*	YIT 4044^{T}	−	*Lactobacillus murinus*	YIT 0239^{T}	−
B. animalis subsp. *lactis*	YIT 4121^{T}	−			

S2 Table

Validation of Bpl190 probe and reference strains used in this study.

++; strong, +; positive, −; negative, w; weak.
